# The Mediating Role of Brand Trust and Brand Love between Brand Experience and Loyalty: A Study on Smartphones in China

**DOI:** 10.3390/bs13060502

**Published:** 2023-06-14

**Authors:** Meng Na, Li Rong, Mohd Helmi Ali, Syed Shah Alam, Mohammad Masukujjaman, Khairul Anuar Mohd Ali

**Affiliations:** 1 Graduate School of Business, Universiti Kebangsaan Malaysia (UKM), Bangi 43600, Malaysiamasuk@ukm.edu.my (M.M.);; 2 Faculty of Management, Xiangtan Institute of Technology, Xiangtan 411101, China; 3 College of Business Administration, Prince Sultan University, Riyadh 11586, Saudi Arabia

**Keywords:** brand attributes, brand loyalty, word-of-mouth engagement, smartphones

## Abstract

Smartphones have become increasingly essential in people’s daily lives. Studying the enablers that influence students’ smartphone buying intentions can inform technology-enhanced learning development, whereas research on brand loyalty and experience is important for marketing strategies. While prior research has acknowledged the importance of brand experience and customer loyalty, little literature has investigated the specific dimensions of brand loyalty and their connection to brand love and trust. This study investigates the effects of brand attributes on loyalty and word-of-mouth engagement in choosing smartphones in China, including the mediating role of brand trust and brand love between brand experience and loyalty. The study adopted a research framework based on the previous literature and tested it empirically. The study adopted a cross-sectional survey method, collecting 369 questionnaires from Chinese students in mainland China. The collected data were analyzed with the help of structural equation modelling by applying AMOS software version 26. The results showed that brand experience had a significant influence on brand trust, brand love, attitudinal loyalty, and word-of-mouth, except for behavioral loyalty. Likewise, the link between brand trust and attitudinal loyalty, behavioral loyalty, and brand love was found to be significant. The effect of brand love on attitudinal loyalty and behavioral loyalty became significant. Additionally, the study confirmed that behavioral trust and brand love significantly mediates the relationship between brand experience–attitudinal loyalty, and brand experience–behavioral loyalty, respectively. The study results provide numerous theoretical and managerial implications to help academicians and practitioners provide better customer and brand relationship management.

## 1. Introduction

In the current competitive business environment, building and maintaining strong relationships between brands and customers is crucial for business success. Cultivating and retaining a devoted clientele is considered to be the primary factor that drives a corporation’s long-term success, as it reduces marketing costs, enhances competitiveness, boosts market share, generates positive word-of-mouth, and creates more opportunities for expansion [1]. To achieve this, brands must focus on establishing and nurturing brand attributes like brand trust, brand love, and brand experience. These brand attributes have become increasingly important as customers seek meaningful and personalized connections with the brands they engage with [2]. Therefore, it is essential for brands to understand and prioritize these aspects to build strong and lasting relationships with their customers. Therefore, understanding the importance of these concepts and their relationship to consumer behavior is critical for marketers to develop effective branding strategies that can lead to long-term success in the marketplace.

Smartphones have become an integral part of daily life, as most individuals carry one with them wherever they go [3]. The purchase of smartphones was on the rise in recent years, as basic mobile phones have become less common in the market [4]. People of various ages use smartphones from different brands, depending on their personal preferences and brand loyalty [5]. Offering the latest technology and convenient portability, smartphones are now used in nearly every aspect of people’s lives, serving as mini-computers [6]. Smartphones are increasingly becoming a ubiquitous technology and studying smartphone buying intentions can provide insight into the adoption and diffusion of technology. This information can be useful for technology companies in developing and marketing new products and services.

The smartphone industry in China has become a major player in the global market, with several of the world’s largest smartphone manufacturers, including Huawei, Xiaomi, Oppo, and Vivo, establishing themselves as significant competitors to established brands like Apple and Samsung [7]. With over 1 billion smartphone users in China [8], largely due to the increasing affordability of smartphones and widespread availability of mobile internet services, the industry’s growth has not been without its challenges, including the US–China trade war and the COVID-19 pandemic impacting global demand (13.2% decline in the first quarter of 2022) for smartphones [9]. However, with the development of new technologies like 5G and the expected continuation of growth in the industry, Chinese smartphone manufacturers are anticipated to become even more competitive in the global market, offering high-quality devices at affordable prices. Huawei has emerged as the leading smartphone manufacturer in China, followed by Xiaomi, Vivo, and Oppo, respectively.

In order to build the consumer–brand relationship and improve performance outcomes, unique and memorable brand experiences have been increasingly important in branding literature during the past ten years [10,11]. Research shows that modern customers make purchases for a wide range of reasons, including the usefulness and benefits [12,13], as well as the enjoyment [12,13] that the product or service will provide [14,15]. Researchers have clearly acknowledged the significance of the brand experience concept in marketing and brand management literature, as seen by the rising amount of work in the field of brand experience. Despite the growing body of literature on the topics of brand equity, brand personality, brand attitude, brand association, and brand value, the phenomenon of the brand experience remains largely uncharted [16]. Karim et al. [17] studied the relationship between brand love, commitment, and trust with loyalty via measuring the level of awareness, without showing a direct link in the case of the Indonesian banking sector.

Despite the importance of brand experience and consumer loyalty, relatively little marketing literature has investigated the elements that connect these two facets of the industry. This research addresses this issue by focusing on two important brand love and trusts mediators on the linkages between brand experience and brand loyalty. Therefore, in comparison to other studies, this one provides a more accurate process model on how and to what extent customers develop brand loyalty [18]. Additionally, prior research often used brand loyalty as a monolithic construct, omitting its nuances [19,20,21], which may disregard its separate yet important dimensions.

Thus, this study diverges from other research and examines the particular dimensions of brand loyalty, namely, the behavioral and attitudinal dimensions. As a result, compared to earlier research, this study provides a more thorough analysis of the brand loyalty aspects. This study proposes a model that is motivated by brand experience, influenced by brand love and brand trust, and culminates in the outcome of word-of-mouth and brand loyalty. This study tackles these significant but under-researched research concerns. This study focuses, especially, on examining two different issues:

(1) Is there a difference in the effects that brand love, brand trust, and brand experience (i.e., sensory, intellectual, and behavioral) have on brand loyalty (i.e., both behavioral and attitudinal)?

(2) Do the connections between having a positive experience with a brand and being loyal to that brand act as a moderating force between brand love and brand trust?

The present study will contribute to the literature, exploring new relationships such as brand experience and attitudinal aspects, behavioral loyalty and word-of-mouth. The manufacturers of smartphones will benefit by understanding young students’ brand loyalty and the related predictors.

The remaining parts of this paper are structured as shown below. After conducting a thorough analysis of the pertinent research, the author next presents the fundamental theory, which is known as the brand resonance model. After that, the method, findings, and conclusions are presented, followed by the study model and the assumptions. In the final section, we discuss the implications for theory and management, we discuss some limitations, and then draw some conclusions about where future research should go.

## 2. Theoretical Background

### 2.1. A Theoretical Perspective on Brand Experience

Using “sensations, feelings, cognitions, and behavioural responses triggered by brand-related stimuli that are part of a brand’s design and identity, packaging, communications, and environments,” Brakus et al. [22] defined brand experience (p. 53). They looked at “experience” from the perspectives of marketing, cognitive science, philosophy, and applied management in order to explain the idea of brand experience. According to Brakus et al. [22], the total brand experience is made up of many experiential notions such as product, shopping, service, and consumption experiences.

According to Hirschman and Holbrook [23], the idea of brand experience places a strong emphasis on the cognitive and emotional components of the consumer process. Schmitt [24] was the first to define brand experience in terms of its social, behavioral, affective, sensory, and cognitive dimensions. Before him, no research had tried to explore or clarify the brand experience in terms of its dimensions (unidimensional or multidimensional). Later, Brakus et al. [22] developed the idea of the brand experience as a multidimensional construct made up of four dimensions: sensory, affective, behavioral, and cognitive, and empirically confirmed this idea. Although it is conceptually separate from emotional, evaluative, and associative brand conceptions, such as attachment to a brand, attitude toward a brand, and affiliation with a brand, the brand experience does share certain parallels with these other types of brand conceptions [22].

Experience, on the other hand, is not an example of an emotional bond and cannot be used to define one. One example of this is brand attachment, which may be regarded as a powerful emotional connection between the customer and the brand [25]. Internal reactions to external stimuli that set off experiences are what lead to the development of an emotional connection between a consumer and a brand over time [22].

According to another definition, brand experience is “the perception of the consumers, at every moment of contact they have with the brand, whether it be in the brand images projected in advertising, during the first personal contact, or the level of quality regarding the personal treatment they receive” (Alloza, [26], p. 373). When a consumer searches for information, buys a product or service, receives it, and consumes it, they are experiencing a brand [10,15,24]. Additionally, Ramaseshan and Stein [11] claimed that several brand cues, including product design, identity, packaging, distribution hubs, and marketing communications, have an impact on how consumers view a brand. Additionally, studies claim that the brand experience serves as the cornerstone of a consumer’s comprehensive assessment of the brand [27,28]. Prior research has, however, also sought to explain the experience in terms of the “takeaway impression” a customer develops after interacting with a business [29,30]. During the customer’s engagement with the brand, a number of circumstances (antecedents) combine to create these “take-away impressions” and results (consequences). Then, the study went on to describe the brand experience model, which identifies key antecedents and potential consequences of the brand experience.

### 2.2. The Brand Resonance Model

The brand resonance model measures brand strength based on consumer perceptions, assumptions, and attitudes. Marketing managers must choose what and how consumers should learn about the brand, what its differences and associations are, the desired reactions, and the reasons why consumers should be brand loyalists [31], as well as what the experience will be with the brand [32], in order to achieve the desired results. The strongest brands may be determined according to their customers, however brands with issues can also be determined by their customers [33].

According to the brand resonance concept, it is possible to achieve brand resonance, such as brand loyalty, using both logical and emotional approaches [34]. The rational route contains aspects, such as performance (for example, price, efficiency, durability, dependability), judgment (for example, quality, credibility), and so on, whereas the emotional route includes aspects, such as imagery and sentiments (for example, delight and excitement) [34]. Because of a variety of factors, making the option to pick brand love and brand trust as the mediators between brand loyalty and each path are made much simpler. The concept of brand trust was selected for this investigation because previous research indicated that it plays an important role as a mediator in the brand loyalty model. Brand love, on the other hand, is a high-order construct that incorporates a variety of sensations, making it an excellent candidate for the role of mediator in our investigation [35]. These two mediators represent the logical and emotional aspects of brand relationship quality (BRQ) [36], respectively.

### 2.3. Conceptual Model and Hypotheses Development

#### 2.3.1. Relationship between Brand Experience (BE) with Brand Trust (BT)

Customers have a variety of expectations when they buy a branded product [37]. Customers trust companies that live up to their expectations [38]. According to Loureiro et al. [39], corporate characteristics, consumer-brand traits, and brand characteristics are the three main antecedents of brand trust. Brand trust is impacted by all three of the brand experience components, both separately and together. According to Hwang et al. [40], experience–trust relationships are crucial in both the service and non-service industries. Previous studies have shown that satisfiec customers exhibit long-lasting engagement and trust in the brand [41]. In a similar vein, it was discovered that customers who have a negative brand experience do not trust or return to that brand. Additionally, customers may discuss their unpleasant interactions with these brands [42]. Brand characteristics and consumer personality factors both affect consumer trust in a product or service. Additionally, a company’s participation in corporate social responsibility improves its brand image [43]. According to Gentile et al. [44], customers should have faith in companies if they have previously had a positive encounter with them. As a result, customers and the brand may establish a long-lasting bond. Heinrich et al. [42] and Berry et al. [45] both reported findings like similar by Gentile et al. [44]. Huaman-Ramirez and Merunka, [46] found a strong relationship between brand experience and brand trust. On the basis of the discussion above, we developed the following hypothesis:

**H1:**  *BE significantly affects BT.*

#### 2.3.2. Relationship between Brand Experience and Brand Love (BL)

Customers have specific expectations when they buy branded goods. Users of the brand will like their interactions if the supplied value exceeds their expectations. Additionally, Khan and Rahman, [27] and Fullerton [47] highlighted that relationships and feelings toward the brand help to improve the perception of the brand by consumers. Brand love is the term used to describe a customer’s sustained emotional connection to a brand. According to Carroll and Ahuvia [48], the level of emotional attachment a satisfied consumer has for a specific brand is often referred to as brand love. Albert et al. [49] and Aron and Aron [50] postulated that the self-inclusion theory postulates that people form social connections with peers and friends because they anticipate receiving attention from others. Therefore, it is thought that customers are in love with a brand when they form a long lasting and emotional bond with it. Similar to this, Albert et al. [49] emphasize how loyal a brand’s relationship with its customers is. Customers view the brand favorably as a result of this connection. Khan and Rahman [27] and Wallace et al. [51] both contend that customers who are pleased with a brand’s value proposition have a strong emotional bond with it. Because of this, these customers are more likely than others to pay greater rates. Additionally, customers may not be interested in other brands and are not price sensitive when it comes to the brand they love. Arora [52] revealed that consumers experience anguish if they are unable to purchase the brand they love. Numerous qualitative research has shown that customers’ emotional and cognitive experiences with a company promote brand love and improve its reputation and loyalty [51]. Additionally, it was asserted that consumers’ favorable interactions with a brand promote happy emotions and raise their satisfaction levels [53]. Additionally, Sathish and Venkatesakumar [54] underline that brand love is preceded by a consumer’s emotional results, such as happiness, intimacy, and affective feelings for a brand. Accordingly, we proposed the second hypothesis:

**H2:**  *BE significantly affects BL.*

#### 2.3.3. Relationship between Brand Experience and Loyalty

As separate from services as products are from services, experiences are a unique economic offering. The experience is a real offering, just like any other service, good, or commodity; it is not some abstract concept. An experience occurs when a business purposefully uses services as the props to draw certain customers, in a way that produces an unforgettable event [13]. However, prior research on experiences has focused more on practical product features and category experiences than on brand-specific experiences [22]. Brand-related stimuli that are an element of a brand’s design and identity, packaging, communications, and settings were identified as evoking subjective, internal consumer reactions and behavioral responses [22]. The degree to which customers use a brand, talk about a brand with others, look for brand information, promotions, events, etc., was also taken into account [55]. The strength and intensity of brand experiences varied, that is, certain brand experiences were stronger or more intense than others. Like product experiences, brand experiences varied in valence, meaning that some were more positive than others and some could even be unfavorable. Additionally, while some brand experiences happen quickly and without much thought and are brief, others happen consciously and are more sustained. These enduring brand memories that customers retain over time ought to affect their happiness and loyalty [22,56]. According to other researchers, the brand experience was essential to retaining customers’ loyalty [13].

Brand experience, according to certain earlier studies [47,57,58], has an impact on brand loyalty. Additionally, earlier research revealed that brand experience was a predictor of both behavioral and attitudinal loyalty [59,60]. Prior research has shown that brand experience positively influenced both attitudinal loyalty (AL) and behavioral loyalty (BHL) [60,61]. A recent study by Cuong [62] confirmed that brand experience has a significant effect on brand loyalty. Accordingly, in this study we proposed that:

**H3:**  *BE significantly affects AL.*

**H4:**  *BE significantly affects BHL.*

#### 2.3.4. Relationship between Brand Experience and Word-of-Mouth (WM)

The second result on brand experience that this research identified is a person’s propensity to share favorable brand messages through word-of-mouth. “Informal, person-to-person communication between a perceived non-commercial communicator and a receiver regarding a brand, a product, an organisation, or a service”, is how Harrison-Walker [63] describes word-of-mouth (p. 63). Consumers interact with other people they are connected to, through formal or informal groups, by sharing their brand experiences with them [64]. Individuals in these groups converse and share thoughts, suggestions, and opinions [65]. In a number of scenarios, studies have identified word-of-mouth as a result of the brand experience (see [66,67]).

According to Iglesias et al. [68], brands that offer a superior customer experience can foster a greater emotional connection with their customers. The correlation between positive customer experiences and strong branding is well established, as shown by Alexandrov et al. [69] and Mukerjee [70], who suggest that brand experiences influence word-of-mouth marketing. Likewise, Khan and Fatma [16] established that brand experience significantly and positively affects word-of-mouth referrals. Serra-Cantallops et al. [71] highlight that companies can become brand promoters and value co-creators by offering unique and memorable experiences. This can result in positive recommendations from satisfied customers. As a result, this study suggested that:

**H5:**  *BE significantly affects WM.*

#### 2.3.5. Relationship between Brand Trust and Brand Love

A brand’s ability to anticipate or explain its customers’ behavior and provide high levels of satisfaction can be directly tied to its ability to meet their emotional demands, as stated by Ahuvia and Ahuvia [48], who argue that this is the case when a brand establishes and maintains a long-term trading relationship with its customers. In line with Sternberg’s triangle theory of interpersonal love [72], the concepts of “brand commitment,” “brand closeness,” and “brand enthusiasm” are used to measure brand love. We argue that a customer’s contentment with a brand might rise to the level of love when they come to perceive that brand as an extension of themselves [48,73]. Customers who have faith in a brand are more likely to make a purchase because they have confidence in the firm and the claims made by the brand [74,75]. Despite the lack of clarity, Chaudhuri and Holbrook [76] argue that consumers’ emotional responses to and confidence in a brand are both crucial factors in determining their loyalty to that brand.

Trust in a brand can be influenced by consumers’ emotional responses to that brand, according to a study by Song et al. [77]. Brand trust has a positive effect on brand love [35,78]. Brand love, in turn, is influenced positively by brand trust. The connection between brand trust and brand love has recently been established by research conducted by Zhang et al. [79]. As a result, the following hypothesis was put forth:

**H6:**  *BT significantly affects BL.*

#### 2.3.6. Relationship between Brand Love and Brand Loyalty

Customers do not decide what to buy at random. Instead, they are influenced over time by a number of internal factors. Customers will be psychologically connected to a brand, in addition to their recurring buying habits. This means that brand loyalty not only draws in new customers, but also keeps them coming back for more in a cutthroat market. The majority of empirical studies suggest that in order to accurately measure brand loyalty, both attitude and behavior must be taken into account, i.e., behavioral loyalty and attitudinal loyalty [80,81,82,83]. Consumers that have an attitude of loyalty to a brand are more likely to buy from them and suggest their goods and services to friends, family, and other people, even if the price is greater. The degree to which a customer prefers a branded good or service, that is, how likely they are to use that good or service again, is referred to as behavioral loyalty. Brand love is a result of brand jealousy rather than directly influencing consumers’ desires to make a purchase. According to Barbara and Ahuvia [48], consumers who have a strong emotional connection to a brand are more likely to recommend it to others and are willing to pay a higher price for it [75,76]. According to Chen and Quester [84], Batra et al. [85], and Barbara and Ahuvia [48], brand love also influences brand loyalty, while the recent study by Izquierdo-Yusta et al. [86] found a significant relationship between BL and BHL. Similarly, the study by Song and Kim, [87] proved that BL influences AL significantly. So, we proposed that:

**H7:**  *BL is significantly linked to AL.*

**H8:**  *BL is significantly linked to BHL.*

#### 2.3.7. Relationship between Brand Trust and Brand Loyalty

Customers’ judgments on a brand’s dependability and/or responsibility for their welfare are what is meant by brand trust, which is why it contains a cognitive component [88]. From a conceptual standpoint, people who trust a brand and feel secure with it are more likely to make a purchase in the near future or to exhibit behavioral loyalty toward it. Brand trust is closely tied to attitudinal loyalty due to its ability to elicit positive emotions, lower perceived risk, generate word-of-mouth recommendations, create a sense of identity, and foster shared values. Consumers are more inclined to remain loyal to a brand that makes them feel good, and trust plays a critical role in evoking these positive emotions [76]. When consumers have faith in a brand, they feel less apprehensive about doing business with it, resulting in a greater likelihood of attitudinal loyalty since they are more comfortable and confident in making purchasing decisions [2]. Additionally, when consumers trust a brand, they often develop a sense of affiliation and belonging to that brand, resulting in a stronger emotional connection to the brand and increasing attitudinal loyalty. Studies in the past [2,89] have empirically demonstrated the strong relationship between brand trust and attitudinal loyalty. Based on this evidence, the hypothesis was formulated as follows:

**H9:**  *BT is significantly linked to AL.*

Behavioral loyalty refers to the extent to which consumers repeatedly make purchases from a brand over an extended period [90]. Brand trust is closely linked with behavioral loyalty as it fosters repeat purchases, cultivates a sense of dependability, increases customer lifetime value, and generates positive word-of-mouth. When consumers trust a brand, they are more likely to continue buying from it over time because they believe in the brand’s ability to meet their expectations and deliver on its promises [89]. In the same way, when consumers trust a brand, they view it as a reliable source of products or services. This feeling of reliability encourages consumers to come back to the brand again and again, as they know they can rely on the brand to provide consistent quality and value. Moreover, when consumers trust a brand and exhibit behavioral loyalty, they become more valuable to the brand over time [2]. This is because they continue to make purchases from the brand, even as competitors enter the market or offer similar products or services at a lower price. Past studies [2,89,91] have also confirmed that brand trust has a positive impact on behavioral loyalty. Hence, we proposed the following hypothesis:

**H10:**  *BT is significantly linked to BHL.*

#### 2.3.8. Mediating Role of Brand Trust and Brand Love

This study adheres to the brand resonance model’s rationales, stating that brand loyalty might be attained by starting with brand salience (e.g., brand experience) and, then, going along both the emotional and rational paths of brand love and trust [34]. Additionally, previous studies [37,88,92,93] revealed the mediating variables of BL and BT to customers’ positive responses, such as commitment and self-identity. The links between BE and BL are mediated by the association between BL and BT [35]. The association between brand experience and attitudinal loyalty was widely studied in the marketing literature [59,60], and it was found that positive brand experiences can lead to higher levels of attitudinal loyalty. However, the relationship between brand experience and attitudinal loyalty is not direct, and it is often mediated by brand trust. When consumers trust a brand, they believe that the brand will deliver on its promises and meet their expectations. This belief leads to positive emotions and attitudes toward the brand, which ultimately translate into attitudinal loyalty. In contrast, when consumers do not trust a brand, they are less likely to develop positive emotions and attitudes toward the brand, which leads to lower levels of attitudinal loyalty.

Likewise, brand experience and behavioral loyal were found to be linked in many earlier studies [60,61]. Customers who trust a brand are more likely to repurchase from the brand, recommend the brand to others, and even forgive the brand for occasional mistakes or shortcomings. When customers trust a brand, they are also more likely to perceive the brand as reliable, dependable, and consistent, which further strengthens their loyalty. In contrast, customers who do not trust a brand are less likely to exhibit behavioral loyalty. If a customer has a negative experience with a brand, they are less likely to trust the brand and may even switch to a competitor. Negative experiences can erode trust and decrease the likelihood of repeat purchases and recommendations. Therefore, we proposed that:

**H11:**  *BT mediates the association between BE and AL.*

**H12:**  *BT mediates the association between BE and BHL.*

Furthermore, brand love is a strong emotional connection that customers feel toward a brand, and it is closely related to attitudinal loyalty [87]. When customers love a brand, they are more likely to have positive attitudes toward the brand and be willing to make repeat purchases and recommend the brand to others. Brand love also creates a sense of loyalty and a willingness to overlook occasional mistakes or shortcomings by the brand. In contrast, customers who do not feel brand love are less likely to exhibit attitudinal loyalty. Even if they have positive experiences with a brand, they may not feel a strong emotional connection to the brand and may be more likely to switch to a competitor.

Similarly, brand love creates a strong emotional connection with customers, and it motivates them to engage in behaviors that are beneficial to the brand [86]. Customers who feel brand love are more likely to repurchase from the brand, recommend the brand to others, and even defend the brand in the face of criticism or negative publicity. Brand love also creates a sense of loyalty and a willingness to overlook occasional mistakes or shortcomings by the brand. In contrast, customers who do not feel brand love are less likely to exhibit behavioral loyalty. Even if they have positive experiences with a brand, they may not feel a strong emotional connection to the brand and may be more likely to switch to a competitor. Thus, we proposed that:

**H13:**  *BL mediates the association between BE and AL.*

**H14:**  *BL mediate the association between BE and BHL*

**Figure 1:**
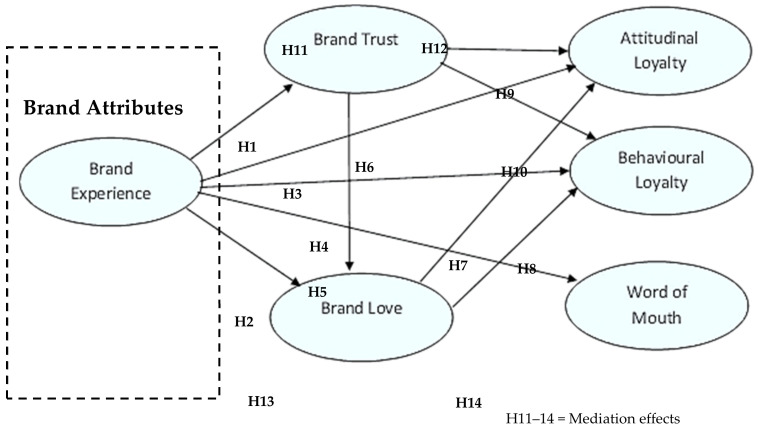
Conceptual framework.

Figure 1 shows the hypothetical relationship between variables.

## 3. Methodology

### 3.1. Research Design

The present research is a quantitative study conducted in China. An empirical survey was conducted to accomplish the objective of the current research. Some methodological issues are discussed in detail in the following sections.

### 3.2. Sample and Method

The present study employed the cross-sectional survey method to gather data using a questionnaire survey method from Chinese Universities. The sample frame was university students in China. The duration of the survey was for 2 months from March to April 2022. Smartphone brands such as Huawei, Apple, Xiaomi, Vivo, OPPO, Samsung, and OnePlus were chosen for this study, as those brands are popular in the Chinese market [94].

This research used the a priori test created by the G*power software to determine an appropriate sample size. As per Cohen’s proposal [95], the recommended sample size was 146 for 6 structures or predictors (F^2^ = 0.15 for effect size, = 0.05 for error type one, and = 0.20 for error type two). A good rule of thumb for sampling, according to Barclay et al. [96], is to multiply 10 by the largest number of formative indicators used. With 21 questions, this study required a total of 210 participants. Yet, to lessen the potential problems from a tiny sample size, we approached 410 respondents, although just 369 questionnaires were found to include correct responses in all sections and the other 41 questionnaires were incomplete. The non-probability convenience technique was used in this study. It was a feasible option because of the expenses and comfort it offered in acquiring enough respondents. We conducted an on-campus in-person questionnaire survey method. Students were selected with the help of their course teachers and with the help of their respective class representatives for choosing students in class. For picking smartphone brands we obtained informed consent by taking signatures from each of the respondents by disclosing the academic aim of this research and how it could contribute to business operations and academia. We also guaranteed to them that their information would not be disclosed individually and that the processed information would be used for publication, so that their individual rights would not be violated.

The study utilized a back-translation technique to ensure the accuracy of the survey. Here is how it was carried out: Firstly, the research questionnaires were thoroughly reviewed by three academicians. Secondly, we sought assistance from proficient research experts who were fluent in both English and Chinese to translate the questionnaire into Chinese. Thirdly, two professional translators who spoke both English and Chinese independently translated the Chinese questionnaire back into English. Fourthly, the quality of the translation was evaluated by comparing the two versions. In instances where discrepancies were found, the researchers and translators collaborated to resolve them. Finally, the questionnaire underwent a pre-test to determine its accuracy, and any necessary modifications were made and confirmed. The Cronbach alpha of the constructs was higher than 0.750, along with each factor loading which was above 0.5. Therefore, the study was found to have the appropriate reliability and validity.

### 3.3. Measures

All items were adopted or adapted from previously validated questionnaires to measure the instruments. Brand experience and brand love were adapted from the scales developed by Kazmi [97]. The measures for brand trust were adopted from the study by Khan [16], and attitudinal loyalty was adopted from Zhang [79]. Brand loyalty was revised and modified from Choi [98]. Moreover, word-of-mouth was modified and adapted from Khan [99]. In the survey, all the questions adopted a six-point Likert scale, which is represented by 1 “strongly disagree”, 2 “disagree”, 3 “somewhat disagree”, 4 “somewhat agree”, 5 “agree”, and 6 “strongly agree”.

### 3.4. Common Method Bias

Based on the suggested guidelines by Harman [100], in this research the common method bias was tested by utilizing exploratory factor analysis. The Kaiser–Meyer–Olkin (KMO) standard was adopted to evaluate the sampling adequacy for factor analysis. The analysis findings demonstrated that all values in the matrix’s diagonal were above 0.5, while the KMO value was 0.933. Moreover, the Kaiser–Guttman standard and the screen test were applied to distinguish the number of existing variables. The evaluation demonstrated that eight factors have more principal eigenvalues than one, which accounted for 68.22% of the variance. In contrast, the primary factor represented 23.2% of the variance in the factors which are lower than 50%. As per the factor analysis, more than one single factor showed up, and most of the variance was not represented by one general factor. Along these lines, this affirmed that there was no presence of common method bias.

## 4. Result and Analysis

### 4.1. Descriptive Statistics Classification

Overall, a larger part of the respondents were male (53.21%); as expected, respondents between 20–25 years of age (68.66%) were the significant contributors to this research. Graduate students were the larger portion of the total respondents (58.48), while the next group was under-graduate students (47.52) (Table 1).

### 4.2. Measurement Model (Reliability and Validity)

Internal consistency and construct validity were estimated through the evaluation of the measurement model. The AVE (average variance extracted) and composite reliability tests were used to investigate the construct validity. Convergent validity (AVE > 0.5) may be inferred for all constructs in Table 2 (see also [96,101]). Since the square root of AVE was greater on the diagonal than it was for any other construct off diagonal [101], this analysis suggests that AVE is discriminately valid (Table 2).

The results were satisfactory in terms of normality, with no significant deviation from normality. Both skewness and kurtosis were under 3 and 10, respectively [101] (Table 3). Variance inflation factor (VIF) analysis was performed to determine the multicollinearity among the independent variables, as described by Kleinbaum et al. [102]. The results suggest that the VIF is between 1.270 to 2.375, which is much less than 10. Because of this, we can safely say that multicollinearity is not the problem in this investigation.

High CR values above 0.7 are indicative of a reliable model and are generally accepted in the preliminary stages of research [103]. Since the CR is higher than the thresholds established earlier, we can confidently say that the study’s design meets or exceeds those standards. The HTMT value was also assessed for robustness because it was proven to be superior by Fornell–Larcker in a number of scenarios [104]. A HTMT score above 0.85–0.90 indicates a lack of discriminant validity [104]. This study is above the minimum required to draw any conclusion (Table 4). All things considered, these analyses do not raise any red flags about the study’s reliability or validity.

### 4.3. Coefficient of Determination

Santosa et al. [105] suggested that a model’s ability to explain things should be measured by figuring out the coefficient of determination (R^2^) of the endogenous variable. According to Falk and Miller’s [106] calculations, the R^2^ value for the endogenous variable should be 0.10. Cohen [107] suggested, based on the work of other scholars, that an R^2^ of 0.26 for endogenous constructs is considerable, an R^2^ of 0.13 is moderate, and an R^2^ of 0.02 is weak. Table 2 shows that all of the endogenous values estimated to be true in this study have R^2^ values higher than the minimum required by Falk and Miller [106], indicating that the model is within a valid range.

### 4.4. Confirmatory Factor Analysis

Confirmatory factor analysis was used to evaluate the reliability of the measurement model’s factor confirmation (CFA). Table 5 displays the fit indices for the canonical factor analysis (CFA) model that resulted, which are all satisfactory: 2/df = 1.355, goodness of fit index (GFI) = 0.923, Tucker–Lewis index (TLI) = 0.982, IFI = 0.985, comparative fit index (CFI) = 0.985, NFI = 0.945, root mean square error of approximation (RMSEA) = 0.037. All item t-values were significantly lower than 5% (Table 5).

### 4.5. Structural Modelling

**Figure 2:**
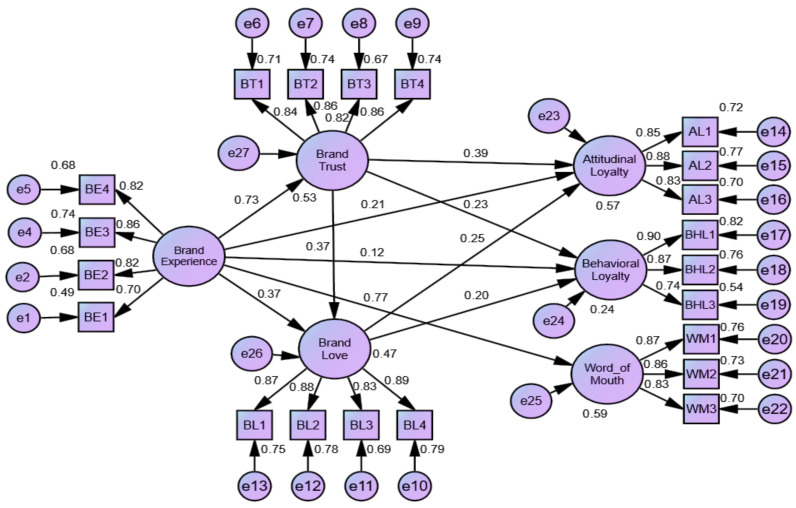
Structural model.

Figure 2 depicts the analytical framework used in this study. The validation of the structural model included a check of the proposed model’s goodness of fit indices. The CFA test of the measurement model’s computation was successfully carried out, therefore this allowed for the validation of the structural model. The results of the structural equation modelling exercise indicated a very good fit between the data and the conceptual framework (χ^2^/df = 1.738) (Table 4). The root mean square error approximation (RMSEA) was calculated to be 0.053, which is below the threshold of 0.08 recommended in the literature [111]. Each of the remaining fit indices were also above 0.9 [112], including the CFI, GFI, IFI, and TLI.

The results (Table 5) show brand experience has a significant influence on the BT (β = 0.725; t = 9.692), BL (β = 0.367; t = 4.191), AL (β = 0.210; t = 2.449), and word-of-mouth (β = 0.768; t = 10.289), except for the BHL (β = 0.122; t = 1.169). Likewise, the AMOS output (Table 6) displays that the link between the BT and AL (β = 0.391; t = 4.527), BHL (β = 0.229; t = 2.204) and BL (β = 0.370; t = 4.348) are significant. The effect between the BL and AL (β = 0.247; t = 3.421), and BHL (β = 0.200; t = 2.257) becomes significant. Therefore, we accept hypotheses 1 to 10, which were found significant at a 1% level of significance except hypothesis 4, which is rejected in this investigation.

### 4.6. Mediation Effect of Behavioral Trust

As proposed by [113], the current study used the Sobel test to examine the mediation effect. Due to the normally distributed nature of the data, we used the Sobel test rather than the bootstrapping technique. To get the necessary support, it is important to undertake the right analysis for the joint significance of the indirect effects technique. The study confirms that BT mediates the relationship between BE and AL (β = 0.283, t = 4.101, *p* < 0.05), and BE and BHL (β = 0.166, t = 2.149, *p* < 0.05) significantly. It is clear that BE and AL (β = 0.090, t = 3.847, *p* < 0.05), and BE and BHL (β = 0.073, t = 1.987, *p* < 0.05) are significant. Therefore, we accept hypotheses 11 to 14.

## 5. Discussion

The study attempted to integrate brand attributes, such as brand trust, brand experience and brand love, and tested the relationship with loyalty (such as attitudinal loyalty and behavioral loyalty) and word-of-mouth engagement. The proposed framework contained 14 hypotheses, where 13 were accepted. With this, the explanatory power was moderate to high as the R^2^ value for attitudinal loyalty, word-of-mouth, and brand trust were 0.57, 0.59, and 0.54, respectively, while behavioral loyalty and brand love were 0.24 and 0.47, respectively. These results showed that the proposed model is generally comprehensive, adequate, accurate, and functional for understanding brand loyalty and word-of-mouth.

A brand experience is a sort of experiential marketing that includes a comprehensive set of circumstances developed by a business to affect the perception a customer has of a specific product or brand name. Companies try to establish a broad atmosphere of goodwill, dependability, or trust through a mix of the many channels consumers use to interact with a brand, in order to establish a connection between the brand and a certain need or emotion. Hypothesis 1 assumed that BE is related to BT. The result of this study confirmed the hypothesis in line with the past study by Huaman-Ramirez and Merunka [46]. This signifies that a consumer’s positive experience with a particular brand enhances the consumer’s trust. Likewise, hypothesis 2 postulated that BE influences BL. The present study endorses the assumption with the investigation in line with the previous studies [52]. This indicates that when customers find the brand is good in use or as per their expectations, they start liking the brand. A similar result was found for hypothesis 3, where the study claimed that a brand which provides a favorable experience leads to attitudinal loyalty. The result of the study also supports the claim positively and complies with earlier studies [61]. This means that peoples’ positive experience with a brand results in loyalty to the brand, even though it is out of stock or selling at marked-up prices.

Surprisingly, hypothesis 4 did not support the assumption that BE is positively linked with BHL. This is opposite to past studies [61], which found a relationship between the same aspects. BHL indicates the continued intention to buy or the auto-choice of buying. This meant that respondents would not repeat a purchase from their solo experience. There may be other external factors (social influence) which are required for conquering the customer’s mind. Otherwise, the model expects indirect relationships such as mediating variables or moderating variables between experience and behavioral loyalty. In our study, we found evidence of a mediating relationship between brand trust in the association between brand experience and behavioral loyalty (H12). The full mediation result implies that the greater the brand trust in customers’ minds the greater the chance of customers continuing to buy due to the respective positive brand experience. That is, brand trust must be established if we want to ensure BHL through the subsequent brand buying experience. On the contrary, BE is found to have a positive connection to word-of-mouth (H5). This identifies that customers may endorse the brand to others as favorable. Existing studies [89,90] have also accentuated this result in the same manner.

As expected, the relationship between BT and AL is proven as significant in this investigation (H6). The past result by Huang [114] also supports this result. This suggests that the higher the BT in the consumers’ minds, the higher the propensity to establish AL, i.e., waiting or buying with an inflated price. Similarly, the assumption that BT induces BHL (H7) complies with the study by Huang [114]. Our study supports this claim and confirms that the relationship is stronger than other factors with BHL. This infers that the greater the BT for the products, the greater the tendency to generate BHL among respondents. Moreover, this is true for brand love, which was supposed to be related to BT in this study (H8) and which was successfully established as it was proven to be significant. This outcome is similar to the previous study by Zhang et al. [79].

The understanding could be that BT brings BL into the consumer’s mind. Additionally, we assumed that brand love positively influences the AL and BHL of consumers toward the brand (H9 and H10). Both the hypotheses were proven to be true in this regard and also in supporting the past literature by Huang [35]. People with boosted love towards a product brand reflect upon their AL and BL, meaning they wait to purchase and continuously select the brand.

Customers are able to develop certain feelings toward a good brand, such as happiness, amazement, anger, disappointment, or other feelings, when they are confident that their chosen brand can satisfy both their needs and wants. When customers have faith in a brand, this condition can also ignite customer loyalty in the form of attitudes and behaviors.

To better understand consumer behavior, many studies have looked into brand loyalty. It can assist businesses in boosting their earnings and market share. Since the 1990s [115], BT research has been conducted on the subject of consumer behavior. Numerous researchers [116] are exploring the relationship between BT and consumer behavior, such as brand loyalty. There are, however, only a few pieces of research that concentrate on the mediating role of brand trust in the connection between the brand experience and client brand loyalty [117].

Regarding the mediation relationships, the current study found all four mediating factors significant. Hypothesis H11 postulated that BT mediates the relationship between BE and AL. The result confirms that the indirect relationship of BT is partially significant. This means that brand experience also predicts AL via brand trust in product buying choice. Likewise, brand love, which was found to have a direct relationship with AL and BHL earlier, was also found to have a moderating role between BE and both loyalties. This helps estimate that enhanced BL could assist in predicting the association between product BE and brand loyalties indirectly as well (H13 and H14). However, BT and BL as mediator constructs in the association between BE–BHL are evident in the full relationship.

## 6. Conclusions

The purpose of the study was to identify the effects of brand attributes on loyalty and word-of-mouth engagement in choosing smartphones among Chinese students in mainland China. The study also investigated the mediating effect of BT and BL between BE and loyalty. The results showed that BE had a significant influence on BT, BL, AL, and WM, except for BHL on the same. Likewise, the link between BT and AL, BHL and BL was found to be significant. Also, the effect of BL on AL and BHL became significant. Furthermore, the study found that BT mediates the relationship between brand experience and AL and BHL, indicating that the positive impact of brand experience on these two aspects of consumer behavior is partially explained by the influence of BT. This highlights the importance of building brand trust and credibility in order to influence consumer attitudes and behaviors. However, these findings highlight the importance of creating positive brand experiences to improve consumer behavior and increase word-of-mouth for smartphone companies. Overall, the study contributes to our understanding of consumer behavior and provides valuable insights for marketers looking to enhance brand experiences and leverage them for better outcomes. The following are the details on the implications, limitations, and future research directions:

### 6.1. Implications of the Study

#### 6.1.1. Theoretical Implications

Customers can develop enduring relationships with brands when they have favorable impressions of those brands. This connection increases consumer affection, trust, and loyalty to the brand. This study’s findings, which show the value of brand management in the context of service-led reasoning, show that the various types of brand relationships have variable effects on brand loyalty and brand trust. This demonstrates that brand trust and brand love models should be included to increase brand loyalty in addition to enabling customers to develop an intimate psychological relationship with the brand in order to enable them to form a long-lasting emotional connection with it. Generally speaking, our analysis results of this study in the context of smartphone usage offers empirical support for the conceptual framework proposed in this study.

Zhang et al. [79] conducted their research in a comparable fashion, but they did not take into account the word-of-mouth and BE constructs, in addition to the common components that were discussed earlier. According to the findings in this study, different aspects of brands have diverse effects on aspects such as BL, BT, and WM engagement. This finding highlights the significance of brand management in the context of the service-led rationale. This demonstrates the importance of allowing customers to develop an emotional connection to a brand through the incorporation of BT and BL models, which serve to strengthen BL and foster a more personal understanding between the company and the consumer. In general, when we look at brand attributes like brand experience through the lens of service-led logic, we can help firms figure out how to encourage long-term behaviors and attitudes. We can also back up Zhang et al.’s [79] study with data.

BT and BL have been discussed in the literature [77], but their mediation role has rarely been empirically verified. This study provides additional evidence that there are other variables that act as mediators in the relationship between brand loyalty and BL [118,119,120]. Specifically, the study highlights brand love as one of these mediators. Trust in the brand and affection for the brand are essential bridges that lead to an emotional connection with the brand and brand loyalty. These significant outcomes can be applied to extend the currently accepted brand theory and provide support for the suggestions made by Barbara and Ahuvia [48], as well as Heinrich et al. [75]. The entire research model can be improved in order to improve forecasts on brand loyalty and to overcome inconsistent findings on BT when BT and BL are incorporated into the model.

Most earlier studies’ theoretical frameworks for brand qualities and relationships relied on interpersonal theories with a Western focus. On the other hand, interpersonal theories that are applicable to China are distinct from those that are applicable in Western countries. It is important for brands, which are tools for developing relationships, to conform to the society in which they operate. It has been discovered that the Chinese are more likely to be influenced by preexisting relationships, whereas Western studies have primarily concentrated on the dynamics between brands. On the basis of these findings, our research constructs a brand attribute model that is founded on indigenous ways of thinking. It also underlines the necessity to concentrate on brand attributes and brand loyalty, both of which are extremely important. The findings from this research not only provide a deeper level of explanation, but also offer practical advice for addressing one of the more challenging aspects of relationship marketing, namely the process of creating brand loyalty in customers.

#### 6.1.2. Managerial Implications

Given these findings, marketers should employ the finest brand architecture and brand portfolio approach to create the greatest brand. The brand’s quality varies depending on the product and the target market. The brand should be created by the seller with the targeted market in mind. If a brand deviates from what consumers anticipated, this will cause them to see it as having inferior quality.

Increasing a customer’s love for a brand can improve their flexibility and positive awareness toward the brand, which in turn increases the customer’s level of pleasure from the brand. Businesses not only need to keep a trustworthy and communicative relationship with their consumers in order to enhance their repurchase rate, but they also need to improve their customers’ brand loyalty in order to keep their existing customers and attract new ones.

Customers who trust a brand are the ones who control their opinions of it, whether they are positive or negative. Additionally, when consumers have faith in a brand, their psychological process, which includes their commitment to and preference for the brand, is altered, and this is reflected in how they feel about the brand. These findings could be applied by marketers to build consumer trust in their brand by providing a solid guarantee, an accessible company and product line, a sustainable service, dependable items, the best value for customers, consistency, and many other factors.

In order to ensure customer loyalty, whether in the form of attitudinal or behavioral loyalty, the brand’s quality should also be maintained and improved by developing a long-term plan for quality improvement, talking and sharing ideas with customers about improving the quality of products and services, comparing products with competitors, giving supreme priority to quality in all plans and procedures, creating a qualified quality assurance team, and many other things.

#### 6.1.3. Limitations and Future Research Direction

The current study is not beyond having limitations. First, this study is regarded as cross-sectional because the data was acquired through the use of a standardized questionnaire; hence, no longitudinal or experimental data were accumulated regarding the customers’ loyalty to smartphones. In light of this, researchers are strongly encouraged to investigate scenarios in which customers react to various brand relationships and to take into account topics relating to love for a brand, trust in a brand, and loyalty to a brand from both qualitative and quantitative perspectives to the extent that data resources allow for such an investigation. Second, we discussed brand trust, brand love and loyalty based on the brand resonance model. Future studies could be conducted from other perspectives, applying other theories to add value to the research. Third, we found no relationship between brand experience and behavioral loyalty, and we found the presence of an indirect relationship with brand trust as a probable reason for this insignificance. However, other factors such as external influences such as peer influence, and self-efficacy variables can be integrated in advanced studies. Fourth, the moderation constructs are ignored in this study, where future studies could come up with such variables to enrich the literature on brand loyalty, or so. Finally, this study relied on a student sample and the findings may not generalize to other populations, such as non-student adults or children. This can limit the external validity of the study’s findings and make it difficult to apply the results to real-world contexts. Future research could focus on collecting data from a more diverse range of participants, including individuals from different age groups, ethnicities, and socioeconomic backgrounds, to cover such limitations.

## Figures and Tables

**Table 1:** Demographic profile of the respondents.

Demographics	Classification	Frequency	Percentages
Gender	Male	196	53.21
	Female	173	46.79
Age (Years)	<20	89	24
	20–25	253	68.66
	>25	27	7.34
Education	Under-Graduate	175	47.52
	Graduate	216	58.48

**Table 2:** Factor loadings and reliability statistics.

	FL	CA	CR	AVE
**Brand Experience** [97]		0.873	0.878	0.645
BE1: The brand impresses me	0.699			
BE2: I feel happy when I buy this brand	0.822			
BE3: This brand evokes deep feelings in me	0.858			
BE4: This brand provides me with a positive experience	0.824			
**Brand Trust** [16]		0.906	0.909	0.714
BT1: I trust this smartphone brand	0.841			
BT2: This cell phone brand is reliable	0.861			
BT3: This is an honest cell phone brand	0.816			
BT4: This cell phone brand is safe	0.862			
**Brand Love** [97]		0.922	0.924	0.753
BL1: The brand brings me great joy	0.868			
BL2: I like this brand very much	0.882			
BL3: The brand is an absolute delight	0.830			
BL4: I am very attached to this brand	0.889			
**Attitudinal Loyalty** [79]		0.889	0.890	0.730
AL1: I will only use this brand of cell phone	0.849			
AL2: I’d rather buy this brand than others, even if it costs more	0.880			
AL3: I won’t buy any other brand if this one is out of stock	0.834			
**Behavioral Loyalty** [98]		0.874	0.878	0.707
BHL1: I intend to keep purchasing cell phones from this brand	0.905			
BHL2: If I need a phone, this brand would be my preferred choice	0.871			
BHL3: I intend to encourage other people to buy cell phones from this brand	0.737			
**Word-of-mouth** [99]		0.888	0.889	0.728
WM1: I have recommended this brand to many people	0.869			
WM2: I have spoken about this brand to my friends	0.856			
WM3: I have said positive things about this brand	0.834			

Note: FL = factor loading, CA = Cronbach alpha, CR = composite reliability, AVE = average variance explained.

**Table 3:** Fornell–Larcker correlation matrix, normality data and R^2^.

	BE	BT	BL	AL	BHL	WM
Brand Experience (BE)	**0.803**					
Brand Trust (BT)	0.631 **	**0.845**				
Brand Love (BL)	0.561 **	0.594 **	**0.868**			
Attitudinal Loyalty (AL)	0.554 **	0.632 **	0.570 **	**0.854**		
Behavioral Loyalty (BHL)	0.380 **	0.400 **	0.399 **	0.385 **	**0.841**	
Word-of-Mouth (WM)	0.654 **	0.684 **	0.605 **	0.617 **	0.337 **	**0.853**
Mean	3.634	3.564	3.928	3.745	4.421	3.480
Standard Deviation	1.261	1.525	1.656	1.583	1.558	1.610
Skewness	0.684	0.683	0.249	0.315	−0.303	0.734
Kurtosis	−0.337	−1.153	−1.659	−1.397	−1.269	−0.986
R^2^	--	0.53	0.47	0.57	0.24	0.59

** Correlation is significant at the 0.01 level (2-tailed).

**Table 4:** Heterotrait-monotrait (HTMT) and multicollinearity.

	BE	BT	BL	AL	BHL	WM	Tolerance	VIF
Brand Experience (BE)	--						0.483	2.072
Brand Trust (BT)	0.703	--					0.437	2.289
Brand Loyalty (BL)	0.621	0.646	--				0.535	1.870
Attitudinal Loyalty (AL)	0.625	0.598	0.630	--			--	--
Behavioural Loyalty (BHL)	0.433	0.444	0.445	0.436	--		0.787	1.270
Word-of-Mouth (WM)	0.738	0.759	0.670	0.694	0.384	--	0.421	2.375

**Table 5:** Results of CFA and structural model with standards.

Fit Indices	Measurement Values for CFA	Meas. Values for Structural Model	Standards with Sources	
**χ^2^/df**	1.355	1.738	<3	[108]
**IFI**	0.985	0.968	>0.900	[109]
**NFI**	0.945	0.928	>0.900	[109]
**CFI**	0.985	0.968	>0.900	[110]
**GFI**	0.923	0.910	>0.900	[109]
**AGFI**	0.915	0.903	>0.900	[101]
**TLI**	0.982	0.962	≥ 0.90	[111]
**RMSEA**	0.037	0.053	<0.080	[111]

**Table 6:** Structural model and hypothesis testing result.

Hypothesis	STD Beta	STD Error	*t*-Values	*p*-Values	Significance (*p* < 0.05)
H1: BE → BT	0.725	0.095	9.692 ***	0.000	Supported
H2: BE → BL	0.367	0.118	4.191 ***	0.000	Supported
H3: BE → AL	0.210	0.108	2.449 **	0.014	Supported
H4: BE → BHL	0.122	0.151	1.169	0.242	Not Supported
H5: BE → WM	0.768	0.101	10.289 ***	0.000	Supported
H6: BT → AL	0.391	0.085	4.527 ***	0.000	Supported
H7: BT → BHL	0.229	0.119	2.204 **	0.028	Supported
H8: BT → BL	0.370	0.090	4.348 ***	0.000	Supported
H9: BL → AL	0.247	0.067	3.421 ***	0.000	Supported
H10: BL → BHL	0.200	0.095	2.257 **	0.024	Supported
H11:BE → BT → AL	0.283	0.086	4.101 ***	0.000	Supported (Partial)
H12: BE → BT → BHL	0.166	0.112	2.149 **	0.032	Supported (Full)
H13: BE → BL → AL	0.090	0.039	3.847 ***	0.000	Supported (Partial)
H14: BE → BL → BHL	0.073	0.051	1.987 **	0.047	Supported (Full)

Note: BE = brand experience, BT = brand trust, BL = brand love, AL = attitudinal loyalty, BHL = behavioral loyalty, WM = word-of-mouth, *** Significant at the 0.01 level (2-tailed). ** Significant at the 0.05 level (2-tailed).

## Data Availability

The data that support the findings of this study are available from the corresponding authors upon proper request.
